# Cataplexy response with extended-release once-nightly sodium oxybate: Post hoc responder analyses from the phase 3 REST-ON clinical trial

**DOI:** 10.1016/j.sleepx.2024.100109

**Published:** 2024-03-30

**Authors:** Michael J. Thorpy, Clete A. Kushida, Richard Bogan, Akinyemi O. Ajayi, Bruce C. Corser, Jennifer Gudeman

**Affiliations:** Albert Einstein College of Medicine, New York, NY, USA; Stanford University School of Medicine, Stanford, CA, USA; Medical University of South Carolina, Charleston, SC, USA; Florida Pediatric Research Institute, Winter Park, FL, USA; Sleep Management Institute, Cincinnati, OH, USA; Avadel Pharmaceuticals, Chesterfield, MO, USA

**Keywords:** Cataplexy, Narcolepsy, Sodium oxybate, FT218, Once-nightly

## Abstract

**Background:**

Once-nightly sodium oxybate (ON-SXB), an extended-release oxybate formulation, yielded significant (*P* < 0.001 at 6 g, 7.5 g, and 9 g) reductions in cataplexy episodes in participants in the phase 3 REST-ON clinical trial (NCT02720744). This post hoc analysis from REST-ON further characterized changes in cataplexy episodes in participants with narcolepsy type 1 (NT1).

**Methods:**

Participants with narcolepsy aged ≥16 years received ON-SXB (1 wk, 4.5 g; 2 wk, 6 g; 5 wk, 7.5 g; 5 wk, 9 g) or placebo. Percentages of participants with NT1 who had ≥25%, ≥50%, ≥75%, and 100% reductions from baseline in mean number of weekly cataplexy episodes were determined. Two-sided *P* values comparing ON-SXB vs placebo were calculated with Fisher exact test.

**Results:**

Participants with NT1 (ON-SXB, n = 73; placebo, n = 72; modified intent-to-treat population) had a baseline mean number of weekly cataplexy episodes of 18.9 (ON-SXB) and 19.8 (placebo). Of participants receiving the highest doses of ON-SXB (7.5 and 9 g), approximately half had a 50% reduction, one-third had a 75% reduction, and one-tenth had a 100% reduction in their cataplexy episodes vs placebo. Significantly greater proportions of participants receiving ON-SXB vs placebo had respective reductions in weekly cataplexy episodes of ≥25% at weeks 1 (4.5 g; *P* < 0.05), 3 (6 g; *P* < 0.001), 8 (7.5 g; *P* < 0.001), and 13 (9 g; *P* = 0.001).

**Conclusions:**

A significantly greater proportion of participants receiving ON-SXB vs placebo experienced reductions in weekly cataplexy episodes at all tested doses. Approximately 10% of participants taking the 2 highest ON-SXB doses had complete elimination of their cataplexy.

## Highlights

• Once-nightly sodium oxybate (ON-SXB) is an extended-release oxybate formulation.
• Clinically meaningful cataplexy reduction occurred with ON-SXB 4.5, 6, 7.5, and 9 g.
• Complete resolution of cataplexy was experienced by some as early as week 3.
• ON-SXB is a once-at-bedtime treatment option for adults with narcolepsy.

## 1 Introduction

Narcolepsy is a rare, chronic sleep disorder whose primary symptoms include excessive daytime sleepiness (EDS) and cataplexy [[1], [2], [3]]. People who have narcolepsy with cataplexy (narcolepsy type 1; NT1) describe their cataplexy episodes as embarrassing or, at worse, emotionally oppressive and having a substantial impact on their lives [4]. There are limited options available to treat cataplexy [5,6]. Until recently, pitolisant and twice-nightly oxybate therapy (immediate-release [IR] sodium oxybate [SXB] and calcium/magnesium/potassium/sodium oxybates) were the only approved therapies for the treatment of EDS and cataplexy in adults with narcolepsy in the US [[7], [8], [9]]; IR SXB and pitolisant are approved in the EU [10,11]. The IR oxybate formulations are administered twice nightly, with the first dose taken at bedtime and the second taken 2.5–4 hours later [8,9]. Awakening during the night to take the second dose may compromise treatment adherence and lead to safety concerns [[12], [13], [14]].

The extended-release, once-nightly formulation of SXB (ON-SXB; FT218 [LUMRYZ™ (sodium oxybate) for extended-release oral suspension], Avadel Pharmaceuticals, Chesterfield, MO) received US Food and Drug Administration approval for the treatment of cataplexy or EDS in adults with narcolepsy in May 2023 [15,16].

In the pivotal phase 3 REST-ON clinical trial (NCT02720744) of individuals with narcolepsy, ON-SXB treatment resulted in statistically significant, clinically meaningful improvements vs placebo on all 3 coprimary endpoints: mean sleep latency on the Maintenance of Wakefulness Test (MWT), Clinical Global Impression of Improvement rating, and number of weekly cataplexy episodes at all doses analyzed (6 g [week 3], 7.5 g [week 8], and 9 g [week 13]; all *P* < 0.001) [17]. A post hoc analysis revealed a significant reduction from baseline in the mean number of cataplexy episodes vs placebo starting at week 1 (ON-SXB 4.5 g; *P* < 0.05). This post hoc responder analysis further assessed reductions in weekly cataplexy episodes in participants with NT1.

## 2 Methods

### 2.1 Study design and participants

REST-ON study design and eligibility criteria were previously described [17]. Briefly, REST-ON was a phase 3, multicenter, randomized, double-blind, placebo-controlled trial with a 3-week screening period, a 13-week treatment period, and a 1-week follow-up period. Eligible participants were ≥16 years of age with NT1 or narcolepsy type 2, had a mean sleep latency across 5 naps of <11 minutes on the MWT, and had an Epworth Sleepiness Scale score >10. Participants with NT1 were required to self-report continuing cataplexy for the last 3 months, with a mean of ≥8 weekly cataplexy episodes during screening.

Individuals were randomly assigned 1:1 to receive ON-SXB (1 wk, 4.5 g; 2 wk, 6 g; 5 wk, 7.5 g; 5 wk, 9 g) or placebo. Randomization was stratified by narcolepsy type. Concomitant stable stimulant therapy was permitted. All anticataplectic drugs were discontinued before study entry with a 3-week washout period to account for rebound cataplexy.

The study protocol was approved by the site's institutional review board, and each participant (or legally authorized representative for those aged <18 years) provided written informed consent before participation. Study conduct adhered to the ethical principles of the Good Clinical Practice guidelines, the International Council for Harmonisation guidelines, and the Declaration of Helsinki, in addition to applicable national and local laws and regulatory requirements.

### 2.2 Cataplexy assessments

Participants with NT1 recorded the number of cataplexy episodes as 0, 1, 2, 3, 4, or ≥5 per day in an electronic daily diary. At least 3 entries per week were required for the average to be considered an observation. The mean number of weekly cataplexy episodes was calculated as the number of episodes divided by the number of days with available diary data of valid weeks within that period, then multiplied by 7.

Post hoc assessments of cataplexy response were the percentages of participants who experienced ≥25%, ≥50%, ≥75%, and 100% reductions from baseline in the mean number of weekly cataplexy episodes (ie, responders) at weeks 1 (4.5-g dose), 3 (6-g dose), 8 (7.5-g dose), and 13 (9-g dose). The percentage change from baseline in mean weekly number of cataplexy episodes was also assessed post hoc at weeks 3, 8, and 13.

### 2.3 Statistical analysis

Efficacy was assessed in the modified intent-to-treat (mITT) population (all randomized participants with ≥1 efficacy measurement after receiving the 6-g dose). Two-sided *P* values were calculated using Fisher exact test. A mixed-effects model for repeated measures was used to analyze percentage change from baseline in cataplexy frequency [17].

## 3 Results

### 3.1 Participants

A total of 212 participants were enrolled in REST-ON and received medication (safety population); in the mITT population (n = 190), 145 participants had NT1 (ON-SXB, n = 73; placebo, n = 72). Baseline demographic and disease characteristics were well balanced between groups in the safety population (Table 1).

**Table 1:** Baseline characteristics of REST-ON participants with NT1 (safety population).

Characteristic	ON-SXB (n = 80)	Placebo (n = 82)
Mean age (range), y	32.1 (16–72)	32.2 (16–67)
Sex, n (%)		
Female	55 (68.8)	63 (76.8)
Male	25 (31.3)	19 (23.2)
Race, n (%)		
White	62 (77.5)	62 (75.6)
Black/African American	15 (18.8)	14 (17.1)
Asian	1 (1.3)	5 (6.1)
Other	2 (2.5)	1 (1.2)
Region, n (%)		
US	47 (58.8)	42 (51.2)
Rest of the world	33 (41.3)	40 (48.8)
Median BMI (range), kg/m^2^	27.8 (17.7–71.9)	26.9 (18.1–46.5)
Mean BMI (SD), kg/m^2^	29.3 (8.2)	28.6 (6.7)

BMI, body mass index; NT1, narcolepsy type 1; ON-SXB, once-nightly sodium oxybate.

### 3.2 Cataplexy outcomes

**Fig. 1:**
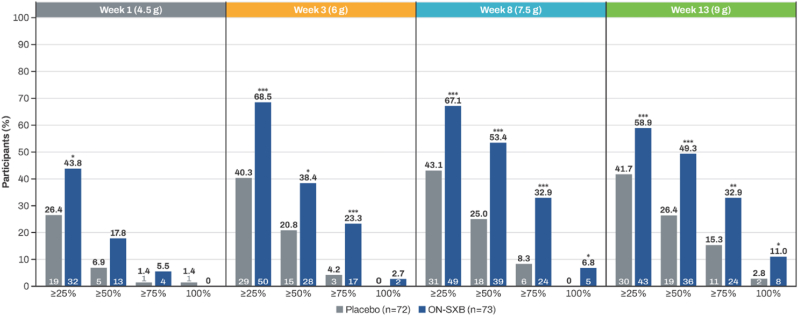
Percentages of participants with ≥25%, ≥50%, ≥75%, and 100% reductions from baseline in weekly number of cataplexy episodes with ON-SXB vs placebo (NT1 cohort, mITT population). mITT, modified intent to treat; NT1, narcolepsy type 1; ON-SXB, once-nightly sodium oxybate (FT218). *P < 0.05, **P < 0.01, ***P ≤ 0.001 (Fisher exact test).

At baseline, the mean [SD] number of weekly cataplexy episodes was similar between treatment arms (ON-SXB, 18.9 [8.7]; placebo, 19.8 [8.9]). During the treatment period, a greater proportion of participants taking ON-SXB had ≥25%, ≥50%, ≥75%, and 100% reductions in the number of weekly cataplexy episodes at all doses compared with placebo (Fig. 1). At the 7.5- and 9-g doses, approximately half of the participants with NT1 taking ON-SXB had a ≥50% reduction in cataplexy episodes (both *P* < 0.001 vs placebo) and approximately one-third had a ≥75% reduction in cataplexy episodes (7.5 g, *P* < 0.001; 9 g, *P* < 0.01). At the end of week 13, cataplexy was eliminated in 11% of participants taking ON-SXB vs 2.8% receiving placebo (*P* < 0.05). Least squares mean change from baseline in the number of cataplexy episodes for participants treated with ON-SXB and placebo, respectively, was −41% and −11% at week 3 (6-g dose), −54% and −20% at week 8 (7.5-g dose), and −62% and −27% at week 13 (9-g dose); the difference (95% CI) in percentage change with ON-SXB vs placebo at weeks 3, 8, and 13 was −30.0% (−42.5 to −17.4), −34.8% (−47.9 to −21.8), and −35.1% (−49.2 to −21.0), respectively.

## 4 Discussion

In these post hoc responder analyses of the phase 3 REST-ON clinical trial, a significantly greater proportion of participants treated with ON-SXB (6, 7.5, and 9 g) experienced ≥25%, ≥50%, and ≥75% reductions in number of weekly cataplexy episodes vs placebo. At the 7.5-g (week 8) and 9-g (week 13) doses, a significantly greater percentage of participants receiving ON-SXB had complete resolution of cataplexy episodes vs placebo (both *P* < 0.05). All evaluated doses of ON-SXB reduced cataplexy ≥25% from baseline vs placebo, meeting the American Academy of Sleep Medicine (AASM) criteria for clinical significance [18]. These findings support the primary data from REST-ON, showing statistically significant improvements in number of weekly cataplexy episodes with ON-SXB (*P* < 0.001 vs placebo) [17].

In the primary REST-ON analyses, comparable efficacy and safety profiles were observed for the 2 highest doses of ON-SXB (7.5 and 9 g) vs placebo, with numerically greater improvement with the 9-g dose (weeks 9–13) [17]. Similar results were observed for the 7.5- and 9-g doses in this post hoc analysis: approximately 10% had complete elimination of cataplexy and approximately one-third and one-half had ≥75% and ≥50% reductions in weekly cataplexy episodes, respectively. REST-ON is the only controlled trial demonstrating efficacy with a 7.5-g dose of oxybate, thus providing evidence to assist clinicians in making dosing decisions for their patients.

REST-ON is also the first clinical trial to show significant reductions in the number of cataplexy episodes after 1 week of SXB treatment (4.5-g dose; *P* < 0.05 vs placebo) [17]. Further clinically meaningful reductions in cataplexy episodes early in therapy were seen in this post hoc analysis, with 44% of participants experiencing ≥25% reductions at week 1. SXB treatment is typically initiated at 4.5 g/night and uptitrated in 1.5-g/night increments weekly until the optimal dose is obtained. These data suggest that patients receiving ON-SXB can expect to have reductions in cataplexy episodes early in titration.

Cataplexy is a burdensome symptom to patients [19], and there are limited approved treatment options [[7], [8], [9], [10], [11]]. Although both SXB and pitolisant are strongly recommended by AASM for the treatment of cataplexy in adults, only SXB is strongly recommended by the European Academy of Neurology; pitolisant is weakly recommended because of moderate quality of evidence due to attrition bias and short trial durations [5,6]. An additional treatment option, particularly one that offers clinically significant improvements in episode frequency earlier in treatment or complete elimination of cataplexy, would be beneficial for patients.

Other treatments evaluating cataplexy have varied in baseline levels of cataplexy. For immediate-release SXB, the first trial enrolled 136 participants with moderate to severe cataplexy with a median of 21 episodes per week [20], which is comparable to the REST-ON baseline value of 19–20 episodes [17].

There are limitations to consider, particularly the post hoc nature of this analysis. In REST-ON, daily episode frequency ≥5 was calculated as 5, which may lead to underreporting of events. The placebo group had an increasing reduction in the number of cataplexy episodes over the 13-week trial, likely reflecting participants understanding that the dose in the active group would increase over time. Despite both these limitations, the observed differences from placebo with ON-SXB were statistically significant in this post hoc analysis, except for 100% reduction at week 3 with the 6-g dose. Additionally, the numbers of cataplexy episodes were not recorded as severe vs mild, or generalized vs local, similar to other prior trials. Therefore, it is not possible to determine if cataplexy severity decreased with ON-SXB treatment. As the impact to people with narcolepsy is greater if generalized, capturing these data in future studies of any cataplexy treatment is needed. Despite this, these findings demonstrate that treatment with ON-SXB significantly reduced the frequency of cataplexy episodes and led to the elimination of cataplexy episodes for some.

## 5 Conclusions

These post hoc analyses on cataplexy reduction corroborate the primary REST-ON results and provide unique insights into the efficacy of a once-at-bedtime dose of ON-SXB as a treatment for cataplexy. These data may provide a useful framework for clinicians when setting expectations of effectiveness for patients with NT1.

## Funding

This study was funded by Avadel Pharmaceuticals (Chesterfield, MO), which was involved in the study design; in the collection, analysis, and interpretation of data; in the writing of the report; and in the decision to submit the article for publication.

## CRediT authorship contribution statement

**Michael J. Thorpy:** Writing – review & editing, Formal analysis. **Clete A. Kushida:** Writing – review & editing, Formal analysis. **Richard Bogan:** Writing – review & editing, Formal analysis. **Akinyemi O. Ajayi:** Writing – review & editing, Formal analysis. **Bruce C. Corser:** Writing – review & editing, Formal analysis. **Jennifer Gudeman:** Writing – review & editing, Formal analysis.

## Declaration of competing interest

Michael J. Thorpy reports a relationship with Axsome Therapeutics Inc that includes: consulting or advisory. Michael J. Thorpy reports a relationship with Balance Therapeutics that includes: consulting or advisory. Michael J. Thorpy reports a relationship with Eisai that includes: consulting or advisory. Michael J. Thorpy reports a relationship with Avadel Pharmaceuticals that includes: consulting or advisory. Michael J. Thorpy reports a relationship with Harmony Biosciences that includes: consulting or advisory. Michael J. Thorpy reports a relationship with Jazz Pharmaceuticals that includes: consulting or advisory. Michael J. Thorpy reports a relationship with NLS Pharmaceuticals that includes: consulting or advisory. Michael J. Thorpy reports a relationship with Suven Life Sciences Ltd. that includes: consulting or advisory. Michael J. Thorpy reports a relationship with Takeda Pharmaceutical Co that includes: consulting or advisory. Clete A. Kushida reports a relationship with Avadel Pharmaceuticals that includes: consulting or advisory. Clete A. Kushida reports a relationship with XW Pharma that includes: consulting or advisory. Richard Bogan reports a relationship with WaterMark Medical and Healthy Humming, LLC that includes: equity or stocks. Richard Bogan reports a relationship with WaterMark Medical that includes: board membership. Richard Bogan reports a relationship with 10.13039/100011096Jazz Pharmaceuticals that includes: consulting or advisory, funding grants, and speaking and lecture fees. Richard Bogan reports a relationship with 10.13039/100007723Takeda Pharmaceutical Co. that includes: consulting or advisory and funding grants. Richard Bogan reports a relationship with Avadel Pharmaceuticals that includes: consulting or advisory and funding grants. Richard Bogan reports a relationship with Oventus that includes: consulting or advisory. Richard Bogan reports a relationship with BresoTec that includes: funding grants. Richard Bogan reports a relationship with 10.13039/100004326Bayer that includes: funding grants. Richard Bogan reports a relationship with 10.13039/501100016198Idorsia that includes: funding grants. Richard Bogan reports a relationship with Suven Life Sciences Ltd that includes: funding grants. Richard Bogan reports a relationship with Balance that includes: funding grants. Richard Bogan reports a relationship with 10.13039/100010902Vanda that includes: funding grants. Richard Bogan reports a relationship with 10.13039/100004334Merck & Co., Inc that includes: funding grants. Richard Bogan reports a relationship with 10.13039/501100003769Eisai that includes: funding grants and speaking and lecture fees. Richard Bogan reports a relationship with 10.13039/100004320Philips that includes: funding grants. Richard Bogan reports a relationship with FRESCA Medical that includes: funding grants. Richard Bogan reports a relationship with 10.13039/100013410LivaNova that includes: funding grants. Richard Bogan reports a relationship with 10.13039/100004337Roche that includes: funding grants. Richard Bogan reports a relationship with Sommetrics that includes: funding grants. Richard Bogan reports a relationship with Harmony Biosciences that includes: speaking and lecture fees. Akinyemi O. Ajayi reports a relationship with Avadel Pharmaceuticals that includes: consulting or advisory. Bruce C. Corser reports a relationship with 10.13039/100011096Jazz Pharmaceuticals that includes: speaking and lecture fees. Bruce C. Corser reports a relationship with 10.13039/100004334Merck & Co., Inc that includes: speaking and lecture fees. Bruce C. Corser reports a relationship with 10.13039/501100003769Eisai that includes: speaking and lecture fees. Bruce C. Corser reports a relationship with Harmony Biosciences that includes: speaking and lecture fees. Bruce C. Corser reports a relationship with Avadel Pharmaceuticals that includes: consulting or advisory and speaking and lecture fees. Jennifer Gudeman reports a relationship with Avadel Pharmaceuticals that includes: employment. If there are other authors, they declare that they have no known competing financial interests or personal relationships that could have appeared to influence the work reported in this paper.
